# Metformin use in pregnancy: promises and uncertainties

**DOI:** 10.1007/s00125-017-4351-y

**Published:** 2017-08-02

**Authors:** Robert S. Lindsay, Mary R. Loeken

**Affiliations:** 1 Institute of Cardiovascular and Medical Sciences, British Heart Foundation (BHF) Glasgow Cardiovascular Research Centre, University of Glasgow, 126 University Place, Glasgow, G12 8TA UK; 2 Section on Islet Cell and Regenerative Biology, Joslin Diabetes Center, Boston, MA USA

**Keywords:** Metformin, Pregnancy, Review, Teratogenesis

## Abstract

Metformin has been prescribed in pregnancy for over 40 years; for much of this time, use has been limited both in numbers and geographically, and the evidence base has been confined to observational studies. In early years, perceived safety concerns and lack of availability of the drug in many countries acted as a barrier to use. More recently, RCTs have begun to examine the role of metformin in pregnancy in much-needed detail. However, this evidence base has been interpreted differently in different countries, leading to very wide variation in its current application in pregnancy. In this short review, we will discuss the history of metformin in pregnancy and highlight some of the key clinical trials. We will then consider some of the remaining controversies associated with metformin use in pregnancy, most important of these being the potential for long-term ‘programming’ effects on the fetus as a result of metformin being able to cross the placenta. We will also consider clinical situations where metformin might be avoided. Finally, we will discuss some future directions for this drug as it reaches its sixtieth anniversary.

**Electronic supplementary material:**

The online version of this article (doi:10.1007/s00125-017-4351-y) contains a slide of the figure for download, which is available to authorised users.

## Early use

The initial development and use of metformin (outside of pregnancy) are reviewed elsewhere in this issue of *Diabetologia* [1]. With regard to pregnancy, it is important to note that it was acknowledged very early on that metformin crossed the placenta. More recent studies show similar plasma concentrations in the maternal and fetal circulation [2]. Further, the combination of increased lactic acidosis risk (mainly observed with the metformin-related biguanide, phenformin) and the relatively hypoxic fetal environment led to important concerns regarding potential adverse effects of metformin use in pregnancy, for both mother and child. In fact, the safety concerns related to phenformin use resulted in the withdrawal of metformin in many, although not all, countries [3].

These early concerns are charted in influential reports of the Aberdeen International Colloquia on Carbohydrate Metabolism in Pregnancy and the Newborn. The first colloquium, reported in 1975, included an entire chapter on ‘the use of sulphonylureas, biguanides and insulin in pregnancy’ [4]. By the time of the fourth report in 1988, the topic of use of metformin was given only a few lines and it was noted that use was not widespread [5].

Metformin use did, however, continue in other parts of the world. In developing countries, the relatively low cost of metformin compared with insulin made it an attractive option. Coetzee and colleagues published a series of important observational papers, commencing in the late 1970s, examining the use of metformin in South Africa [6–8]. In South Africa and other countries, where metformin was routinely used to treat type 2 diabetes, exposure inevitably began to occur in early pregnancy leading to the separate analysis of safety in early pregnancy, particularly regarding miscarriage and congenital anomaly.

However, it was as metformin use became more popular in polycystic ovarian syndrome (PCOS) that a more robust literature developed, investigating exposure of the fetus to metformin in early pregnancy and, therefore, safety of its use.

## Safety and efficacy of metformin during pregnancy

### Safety in early pregnancy

The early literature regarding metformin use in early pregnancy in humans was based on observational findings and of variable quality. Studies were usually small and it was often difficult to tease out the potential teratogenic effects of metformin, particularly as opposed to the well-established effects of maternal hyperglycaemia to increase risk of congenital malformation [9]. Similarly, animal studies have not been completely conclusive, and while increases in embryonic AMP-activated protein kinase (AMPK; AMPK activation being one potential effect of metformin) may be key in diabetic embryopathy, animal studies have not suggested an increase in embryopathy with early metformin exposure in vivo [10].

More recently, a meta-analysis based on metformin exposure in 351 women with PCOS has been carried out. Interestingly, the findings of this study do not suggest an increase in congenital anomaly with metformin use in pregnancy (OR of major birth defect 0.86 [95% CI 0.18, 4.08]) [11]. However, this estimate is based on a small sample size and, therefore, the confidence intervals remain wide.

Metformin has also been extensively analysed in the context of PCOS-associated miscarriage and pregnancy induction. Its use appears to be neutral with regards to miscarriage rates, although some argue that it actually reduces rates of miscarriage [12], and it is superior when used either alone or in combination with clomifene for ovulation induction as compared with placebo [13].

Taken together, while there are important theoretical concerns, metformin appears to be safe in early pregnancy, with no convincing evidence for an increase in congenital malformations or miscarriage with its use. Indeed, in the most common clinical setting of metformin exposure in pregnancy, a woman with type 2 diabetes already on metformin in early pregnancy, the overriding clinical issue should be that discontinuation of use would potentially expose the patient to complications associated with poor glycaemic control. Early clinical assessment and careful consideration of tight control of blood glucose remain key.

### Metformin use in gestational diabetes

As previously mentioned, for some years the use of metformin was limited to specific geographical locations, such as Cape Town in South Africa, but the Metformin in Gestational Diabetes (MiG) randomised clinical trial by Rowan et al in 2008 altered medical practice in many countries [14]. In this study, 751 women with gestational diabetes (GDM) were randomised to either metformin or usual treatment with insulin therapy [14]. The safety profile for mothers appeared to be good. Gastrointestinal side effects led to discontinuation of metformin in 1.9% of women and reduction in dose in 8.8%. The primary outcome included a composite of neonatal hypoglycaemia (<2.6 mmol/l), respiratory distress, need for phototherapy, 5 min Apgar score <7 or premature birth (before 37 weeks), and was no different between the two treatment groups, being present in 32% of both the metformin- and insulin-treated participants. Secondary outcomes, including birthweight, neonatal anthropometrics and rates of large for gestational age (>90th percentile) were also equivalent between the groups. However, the rates of more-severe hypoglycaemia (<1.6 mmol/l) were reduced in the metformin group vs insulin therapy. It is important to note that some 46.3% of women in the metformin group required supplemental insulin treatment to maintain glycaemic control. Metformin appeared to have good patient acceptability, with 76.6% of women suggesting that they would choose metformin in a subsequent pregnancy compared with 27.2% of those initially assigned to insulin. Metformin was associated with less weight gain between enrolment in the trial and 36 or 37 weeks of pregnancy (0.4 ± 2.9 kg in the metformin group vs 2.0 ± 3.3 kg in the insulin group; *p* < 0.001) [14].

More recently, findings from a meta-analysis supported the safe use of metformin as a first-line treatment for GDM (after dietary interventions), showing that this drug has equivalent outcomes to primary insulin treatment with regards to its effects on newborns, and salutary effects on maternal weight gain [15]. Furthermore, this study suggested that metformin may have superior outcomes to the only other oral glucose-lowering agent used in pregnancy, glibenclamide (known as glyburide in the USA and Canada), although only few head-to-head studies were used in the analysis [15]. In support of these findings, a recent small RCT reported a similar safety and efficacy profile of metformin vs glibenclamide [16]. These outcomes are encouraging as, in general, metformin use is less expensive and generally easier for patients to administer than insulin. However, another detailed meta-analysis has also highlighted the limited extent of the evidence base for proper comparison of treatments [17].

Can we select which women are best suited for metformin therapy? Women who required supplemental insulin had a higher BMI in early pregnancy than those maintained on metformin (33.6 ± 8.6 kg/m^2^ vs 31.1 ± 7.8 kg/m^2^, insulin vs metformin), and higher baseline glucose levels (6.1 ± 1.1 mmol/l) compared with those not requiring supplemental insulin (5.3 ± 0.8 mmol/l) [14]. This is broadly similar for other glucose-lowering agents, such as glibenclamide, the failure of which is more likely when initial fasting glucose is high (>6.4 mmol/l) [18], and these observations make sense, a priori, as women with higher glucose levels are likely to have more severe disease. Similarly, women presenting with GDM earlier in pregnancy are more likely to require insulin and may be considered less suitable for oral agents.

### Metformin use in type 2 diabetes

The familiarity of metformin in general diabetes practice and the recent encouraging results from the MiG trial have led to speculation as to its possible uses in women with type 2 diabetes in pregnancy. It is important to note, however, that the evidence base is very small.

Given the insulin resistance of pregnancy, it is likely that most women with type 2 diabetes before pregnancy will require treatment with insulin during their pregnancy, simply to maintain glycaemic control. Is there a role then for metformin as an insulin-sparing agent? There are only a few observational studies on the use of metformin in type 2 diabetes in pregnancy; in 2000, Hellmuth et al reported use of metformin in 50 women in Denmark, 19 of whom had type 2 diabetes [19]. In this retrospective study examining the historical practice with oral glucose-lowering agents between 1966 and 1984, an increase in pre-eclampsia and perinatal mortality was noted with metformin use compared with treatment with sulfonylureas or insulin [19]. Levitt and colleagues reported observational data from South Africa; in their extensive study, women with pregestational type 2 diabetes were treated with insulin or oral glucose-lowering agents before and during pregnancy. Notably, this was also not a randomised study but reflected local practice. Critically, they observed a very high rate of perinatal mortality (125 events per 1000 births) in the group treated with oral glucose-lowering medication (predominantly metformin and glibenclamide) throughout pregnancy compared with women who converted from oral glucose-lowering agents to insulin (28 events per 1000 births) or women who converted from diet to insulin or remained on insulin during pregnancy (33 events per 1000 births) [8]. In contrast, in 2006 Hughes and Rowan reported observational data on the use of metformin in type 2 diabetes in the New Zealand population; they reported no increase in adverse pregnancy outcomes despite a worse risk-factor profile at baseline compared with women not taking metformin [20].

As detailed above, the evidence base for metformin use in pregestational type 2 diabetes is not strong, as trials were not randomised and liable to selection bias. Nevertheless, it would appear inadvisable to consider metformin as a sole agent for the management of women with type 2 diabetes in pregnancy. As a separate issue, there is a potential for use of metformin as an insulin-sparing agent with potential benefits on weight gain in pregnancy and, more, speculatively on glycaemic control. However, there is clearly not enough evidence at this time to recommend such use, with an obvious need for randomised evidence to elucidate the benefits, if any, of metformin as an additional agent for the management of type 2 diabetes in pregnancy. Currently ongoing randomised trials in this area will inform the debate in the future [21].

## Does metformin have long-term effects on the child after treatment in utero?

As detailed above, there is not strong evidence of safety problems for metformin during pregnancy. Therefore, most of the lingering safety concerns revolve around potential long-term effects of metformin exposure in utero. At the time of writing, randomised evidence arising from metformin exposure during pregnancy, to treat GDM, extends to only 2 years, although longer-term studies are expected. In MiG: the offspring follow-up (MiG TOFU), children exposed to metformin in utero were shown to have no difference in total fat mass and body fat percentage, as assessed by bioimpedance, although children did have slightly larger mid-upper arm circumferences, subscapular and bicep skinfolds [22]. There were no differences in blood pressure [23]. Follow-up of a Finnish RCT found that children exposed to metformin in pregnancy were significantly heavier at the age of 12 months, and taller and heavier (12.0 vs 11.3 kg) at 18 months, although the study was small, with a total of 97 children. The mean ponderal index (PI) did not differ significantly and there were no differences in motor, social and linguistic development evaluated at the age of 18 months [24]. Follow-up of a small RCT in women with PCOS suggested no difference in BMI at 8 years, but only included 25 children [25]. The limitations of size of study and length of follow-up in the available studies are noted in a recent review [26].

## Does recent basic science explain how use of metformin in pregnancy can affect the embryo or fetus?

### Is there malformation risk to the embryo?

**Fig. 1:**
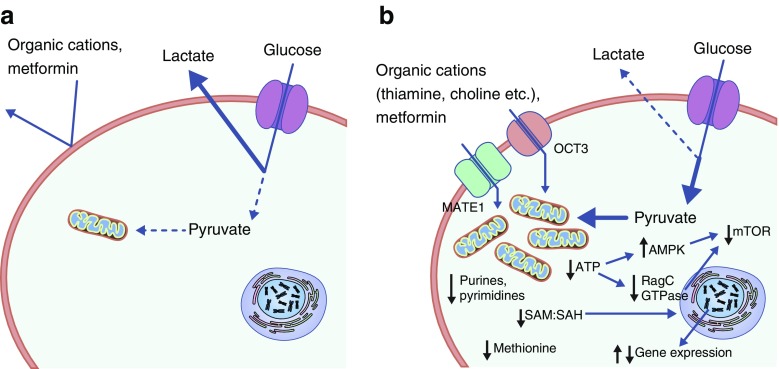
Model showing that susceptibility to metformin depends on the presence of metformin transporters and maturity of mitochondrial function. (**a**) Early post-implantation embryo cells (and, possibly, fetal progenitor cells) are metformin-resistant because of negligible transporter expression. Metabolism is predominantly glycolytic and mitochondrial content is low. (**b**) Differentiated placental ESC (conditioned for high glucose culture). *Caenorhabditis elegans* cells (and, potentially, fetal cells) express higher levels of metformin transporters and are metformin-responsive through effects on mitochondria and gene expression. Metabolism is predominantly oxidative and mitochondrial content is high. RagC, Ras-related GTP-binging protein C; SAH, S-adenosylhomocysteine; SAM, S-adenosylmethionine

As noted above, increased embryo AMPK activity mediates some of the adverse effects of maternal diabetes on congenital malformations in mouse embryos, raising the concern that metformin stimulation of embryo AMPK activity could counteract the beneficial effects of lowering maternal blood glucose levels [27]. In addition, the effects of metformin on one-carbon pathways, which are similar to the effects of anti-folate chemotherapeutic drugs [28], and the essential role of folate in preventing neural tube (and other) malformations [29] raises further concern as to whether metformin might increase embryo malformation risk. However, at doses that stimulated maternal liver AMPK activity, metformin did not stimulate embryo AMPK activity or increase congenital anomalies [10]. Metformin did, however, stimulate AMPK activity and inhibit expression of a gene associated with congenital malformations in mouse embryonic stem cells (ESC) that were used as an in vitro model to study diabetic embryopathy [10]. Interestingly, the difference in metformin susceptibility between mouse embryos and mouse ESC was related to differential expression of metformin transporters [10]. Metformin is transported by organic cation transporters (OCTs) the normal function of which is to take up and extrude organic cations, such as thiamine, choline, neurotransmitters, creatinine, carnitine, guanidine and steroid hormones [30]. *Slc47a1* and *Slc22a3* mRNA, encoding the metformin transporters multidrug toxin and extrusion (MATE) 1 and OCT3, respectively, were expressed at negligible or undetectable levels in embryos, but *Slc22a3* was expressed by ESC, albeit at much lower levels than by maternal liver [10]. The differences in metformin transporter expression and metformin responsiveness between normal mouse embryos and ESC could be a result of metabolic differences between ESC lines in culture and the normal embryo in vivo. It should be noted that the ESC line that was used to study potential teratogenic effects of metformin [10] does not exhibit the responsiveness to high glucose exposure and the high rates of glycolysis relative to oxidative phosphorylation of normal embryos or of ESC derived in physiological glucose media [31]. While conventional stem cell (embryonic and induced pluripotent) lines exhibit fewer, and less mature, mitochondria compared with more differentiated cells, and high rates of glycolysis relative to oxidative phosphorylation are essential for induction of pluripotency from differentiated cells [32–34], conventional ESCs may be more dependent on mitochondrial metabolism (and less dependent on glycolysis) than the normal mouse embryo in vivo. One can speculate that the endogenous solutes taken up by metformin transporters (OCTs) have a function in mitochondrial metabolism, such that metformin transporters would not be expressed by cells that are highly dependent on glycolysis, but would occur upon maturation of mitochondrial respiration (Fig. 1). Consistent with this, metformin and phenformin competitively inhibit thiamine uptake by hepatocytes, causing inhibition of the tricarboxylic acid cycle [35]. Whether the human embryo also lacks metformin transporter expression and metformin responsiveness is not known. However, preimplantation human embryos have low mitochondrial content and are dependent on anaerobic metabolism [32], suggesting that early human embryos may be unresponsive to metformin.

### Are there metabolic programming effects on the fetus?

Compared to the embryo, fetal and placental cells are more differentiated and more dependent on oxidative metabolism and mitochondrial activity. Metformin inhibits complex I of the respiratory chain, leading to an increased AMP:ATP ratio that stimulates AMPK activity [36–38]. AMPK regulates several processes including gene expression and mechanistic target of rapamycin (mTOR)-induced effects on protein synthesis [27, 39–41]. In addition, there are separated, but nonredundant mitochondrial and cytosolic pathways that generate one-carbon intermediates that can be inhibited by metformin, mimicking the ‘methyl folate trap’ that can occur during vitamin B_12_ deficiency [28, 42, 43]. This can lead to methionine deprivation, decreased glutathione (reduced, oxidised disulphide and trisulphide forms), increased homocysteine, and decreased de novo synthesis of purines and pyrimidines [28, 42]. In addition, inhibition of one-carbon metabolism decreases levels of S-adenosylmethionine (SAM) and increases levels of S-adenosylhomocysteine (SAH), which could have epigenetic effects on gene expression because of reduced DNA and histone methylation. Further, a recent study using the round worm *Caenorhabditis elegans* showed that metformin inhibits cell growth and promotes longevity through inhibition of mitochondrial respiration, leading to reduced transit of the Ras-related GTP-binding protein C (RagC) GTPase through the nuclear pore complex (NPC) and inhibition of mTOR signalling [44]. This pathway is highly conserved throughout evolution and may explain the anti-cancer effect of metformin, because the same metformin response reduces viability of melanoma and pancreatic cancer cells [44]. These observations raise the question: since metformin appears to use some of the same mechanisms as nutrient restriction to promote longevity and inhibit cancer growth [42, 45], and dietary restriction (and vitamin B_12_ deprivation) can programme increased cardiometabolic risk in the offspring [46, 47], could metformin exert long-term negative effects on cardiometabolic risk in the offspring?

In order for metformin to affect fetal or placental physiology and development, the cells in these tissues must be able to take up metformin. Furthermore, because metformin is positively charged at neutral pH, there must be a strong mitochondrial membrane potential for it to enter the mitochondrial matrix [48]. Human placentas express several OCT isoforms (OCT1, OCT2, OCT3, and MATE1 and MATE2) [49], and metformin may indirectly affect fetal development, for example, through altered nutrient delivery or placental growth; although, this requires further investigation. Whether fetal tissues express OCTs has not been carefully studied. It should be noted that metformin may not have lasting effects on organs whose growth and repopulation depends on progenitor cells, unless those progenitor cells take up and are metabolically or epigenetically altered by metformin. Yet, in the case of the liver, where it appears that hepatocytes are derived primarily from pre-existing hepatocytes rather than stem cells [50, 51], the epigenetic effects of metformin could have long-term outcomes if chromatin modifications are passed onto daughter cells.
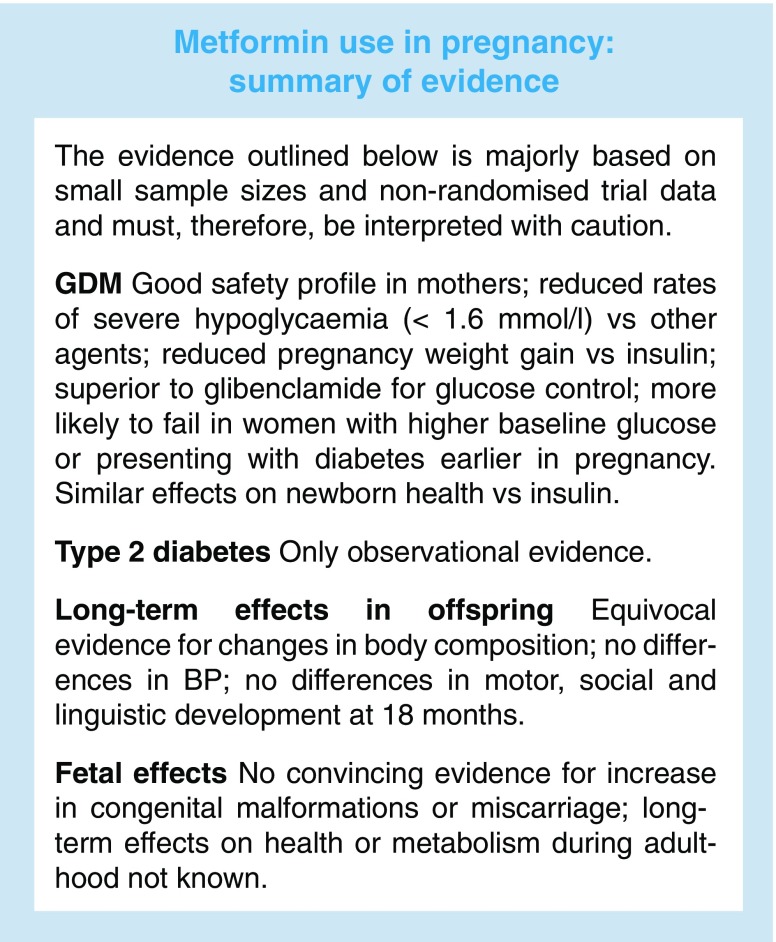


## International use of metformin

Metformin use appears quite varied between countries. In New Zealand, guidelines published by the Ministry of Health in 2014, after extensive review of the evidence, recommended ‘where women who have gestational diabetes and poor glycaemic control (above treatment targets) in spite of dietary and lifestyle interventions, offer oral hypoglycaemics (metformin or glibenclamide) and/or insulin therapy’ [52]. In Scotland, UK, the Scottish Intercollegiate Guidelines Network (SIGN publication no. 116; 2010) suggested that ‘metformin or glibenclamide may be considered as initial pharmacological, glucose-lowering treatment in women with gestational diabetes’ [53].

In contrast, the most recent ADA standard of medical care notes that insulin is the first-line agent recommended for treatment of GDM in the USA [54]. Metformin was noted to have randomised evidence for safety and efficacy, but the lack of long-term safety data for offspring was also noted. The US Food and Drug Administration (FDA) places metformin in category B: ‘Animal reproduction studies have failed to demonstrate a risk to the fetus, and there are no adequate and well-controlled studies in pregnant women’ [54].

In England and Wales (UK), the National Institute for Health and Care Excellence (NICE) recommends that clinicians ‘offer metformin to women with gestational diabetes if blood glucose targets are not met using changes in diet and exercise within 1–2 weeks’. However, at the same time, these recommendations note that the summary of product characteristics for metformin suggests that it should not be used in pregnancy but, instead, insulin should be used [55].

Australian guidelines issued by the Royal Australian College of General Practitioners note that ‘metformin has been used internationally as initial glucose-lowering treatment in women with GDM. However, it has not been approved for this use in Australia for this indication. Lifestyle and insulin therapy remain the mainstay of therapy’ [56].

## Conclusions

International use of metformin in pregnancy has increased greatly in the last decade. The drug is familiar, relatively inexpensive and easy to administer, and is associated with clear benefits as a treatment of hyperglycaemia in pregnancy. For many clinicians, a major concern is the stopping of metformin early in pregestational type 2 diabetes, without adequate replacement, potentially worsening hyperglycaemia. At the same time, as a community, clinicians treating women with diabetes in pregnancy are appropriately, very sensitive to the potential for long-term adverse outcomes. At the time of writing, there certainly do not appear to be clear data suggesting a long-term problem with metformin use, but some clinicians will want to await further data. One troubling aspect of this is that, given the relatively small size of funded studies, difficulty of maintaining cohorts in the longer term, and the potential for high attrition rates, we may never have absolutely clear evidence for this. On its sixtieth anniversary then, perhaps, metformin stands as an exemplar of how research in the management of medical conditions in pregnancy must develop. Those on either side of the debate about its use will agree that better data should have been available earlier and hope that the debate will be able to move on prior to metformin’s next significant anniversary.

## Electronic supplementary material

ESM 1 (PPTX 258 kb)

## Abbreviations

AMPK: AMP-activated protein kinase
ESC: Embryonic stem cells
GDM: Gestational diabetes
MATE: Multidrug toxin and extrusion
MiG: Metformin in Gestational Diabetes
mTOR: Mechanistic target of rapamycin
OCT: Organic cation transporter
PCOS: Polycystic ovarian syndrome
