# Supplementary material for: Metformin use in pregnancy: promises and uncertainties
Source: Diabetologia. 2017 Aug 2;60(9):1612–9. doi: 10.1007/s00125-017-4351-y (PMC5552827; doi:10.1007/s00125-017-4351-y)
Supplement: Supplementary file 1 — (PPTX 258 kb) [file 125_2017_4351_MOESM1_ESM.pptx]

## Slide 1
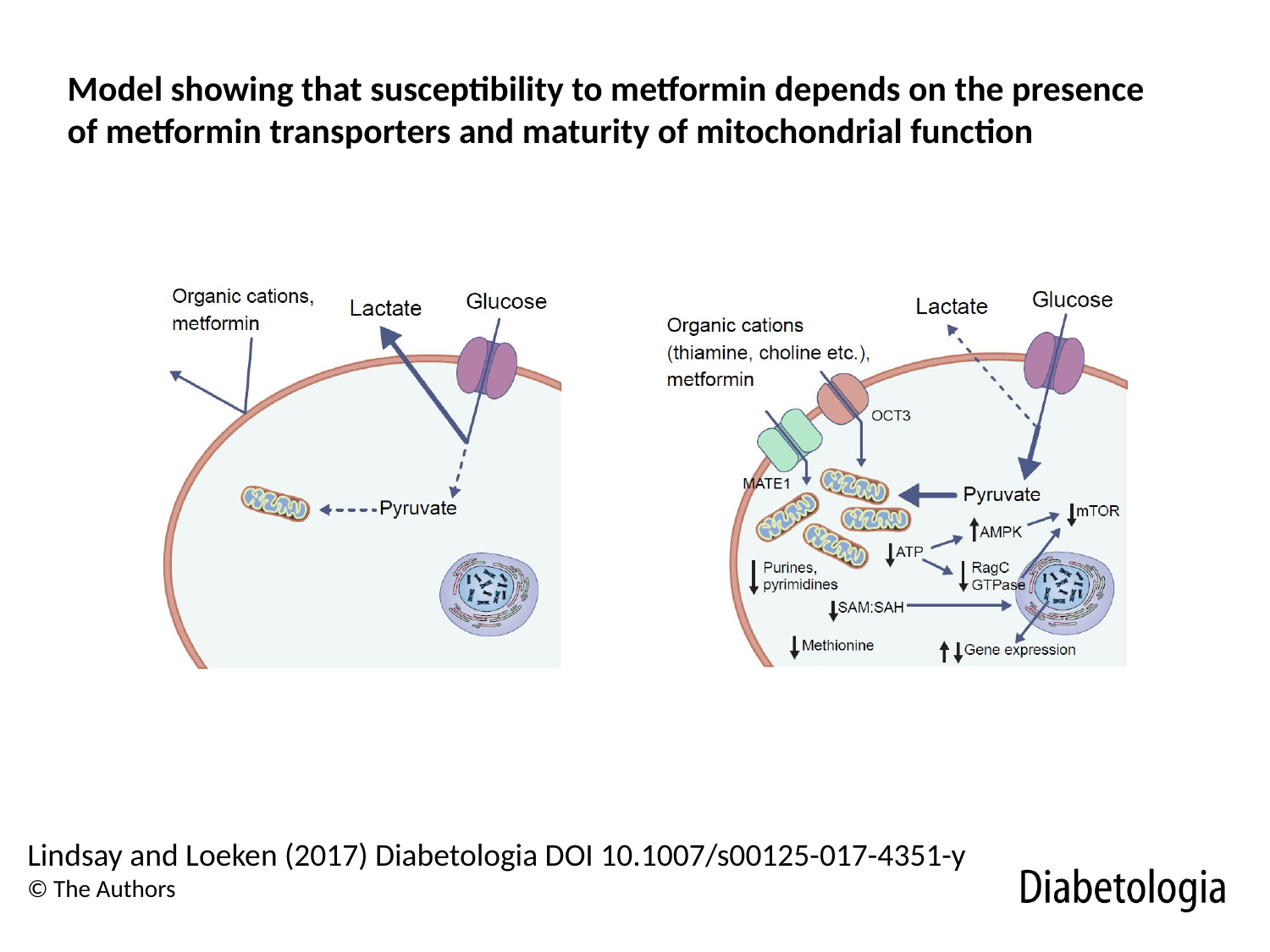

Model showing that susceptibility to metformin depends on the presence of metformin transporters and maturity of mitochondrial function
Lindsay and Loeken (2017) Diabetologia DOI 10.1007/s00125-017-4351-y
© The Authors
